# Proteasome Dysfunction Mediates High Glucose-Induced Apoptosis in Rodent Beta Cells and Human Islets

**DOI:** 10.1371/journal.pone.0092066

**Published:** 2014-03-18

**Authors:** Christophe Broca, Elodie Varin, Mathieu Armanet, Cécile Tourrel-Cuzin, Domenico Bosco, Stéphane Dalle, Anne Wojtusciszyn

**Affiliations:** 1 CNRS UMR 5203, INSERM U661, and Montpellier 1 & 2 University, Institute of Functional Genomics, Montpellier, France; 2 Laboratory for Diabetes Cell Therapy, Institute for Research in Biotherapy, University Hospital St-Eloi, Montpellier, France; 3 B2PE Laboratory (Biology & Pathology of Endocrine Pancreas), BFA Unit, Univ. Paris-Diderot, CNRS EAC4413, Paris, France; 4 Cell Isolation and Transplantation Center, Department of Surgery, Geneva University Hospitals and University of Geneva, Geneva, Switzerland; 5 Department of Endocrinology-Diabetes-Nutrition, University Hospital Lapeyronie, Montpellier, France; University of Lille Nord de France, France

## Abstract

The ubiquitin/proteasome system (UPS), a major cellular protein degradation machinery, plays key roles in the regulation of many cell functions. Glucotoxicity mediated by chronic hyperglycaemia is detrimental to the function and survival of pancreatic beta cells. The aim of our study was to determine whether proteasome dysfunction could be involved in beta cell apoptosis in glucotoxic conditions, and to evaluate whether such a dysfunction might be pharmacologically corrected. Therefore, UPS activity was measured in GK rats islets, INS-1E beta cells or human islets after high glucose and/or UPS inhibitor exposure. Immunoblotting was used to quantify polyubiquitinated proteins, endoplasmic reticulum (ER) stress through CHOP expression, and apoptosis through the cleavage of PARP and caspase-3, whereas total cell death was detected through histone-associated DNA fragments measurement. *In vitro*, we found that chronic exposure of INS-1E cells to high glucose concentrations significantly decreases the three proteasome activities by 20% and leads to caspase-3-dependent apoptosis. We showed that pharmacological blockade of UPS activity by 20% leads to apoptosis in a same way. Indeed, ER stress was involved in both conditions. These results were confirmed in human islets, and proteasome activities were also decreased in hyperglycemic GK rats islets. Moreover, we observed that a high glucose treatment hypersensitized beta cells to the apoptotic effect of proteasome inhibitors. Noteworthily, the decreased proteasome activity can be corrected with Exendin-4, which also protected against glucotoxicity-induced apoptosis. Taken together, our findings reveal an important role of proteasome activity in high glucose-induced beta cell apoptosis, potentially linking ER stress and glucotoxicity. These proteasome dysfunctions can be reversed by a GLP-1 analog. Thus, UPS may be a potent target to treat deleterious metabolic conditions leading to type 2 diabetes.

## Introduction

Type 2 diabetes is characterized by chronic hyperglycemia caused by an impaired function and survival of insulin producing pancreatic beta cells and an insulin resistance of peripheral tissues [1]. Although glucose is the main regulator of beta cell functional mass, it can be deleterious when present in excessive amounts during prolonged exposure [2]. Such a glucotoxicity is responsible for apoptosis of beta cells [3], mainly through inducing an oxidative stress, an endoplasmic reticulum (ER) stress, and an inflammatory reaction. Understanding the exact mechanisms that link glucotoxicity to beta cell apoptosis is crucial for the preservation of a functional pancreatic beta cell mass.

The ubiquitin/proteasome system (UPS) is one of the major degradation pathways for maintaining protein homeostasis. It degrades misfolded, oxidized or damaged proteins, but also regulates proteins involved in many cellular processes, such as signal transduction, cell cycle regulation, cell death, and gene transcription [4], [5]. Proteins to be degraded in the UPS are first tagged with a polyubiquitin chain, then enter the 26S proteasome, a multicatalytic complex that associates a regulatory complex (19S proteasome) with a catalytic core (20S proteasome). The 20S proteasome is a cylindrical structure made of two outer rings of α-subunits and two inner heptameric rings of β-subunits that carry the proteolytic activities, classified as caspase-like (β1), trypsin-like (β2), and chymotrypsin-like (β5), which cleave after acidic, basic and hydrophobic amino acids, respectively [6].

The precise role of UPS in the beta cell remains elusive. UPS regulates key proteins of the beta cell secretory cascade such as K_ATP_ channels [7], voltage-dependent calcium channels [8], or proinsulin [9], thus playing a major but complex role in the regulation of insulin secretion [10], [11], [12], [13]. The role of UPS in the control of apoptosis/survival balance is also controversial [14]. Indeed, UPS regulates key proteins for beta cell survival such as IRS-2, MafA, or CREB [15], but also major regulators of apoptosis such as caspases (review in [16]). High doses of proteasome inhibitors (PIs), that totally block UPS activities, induce severe apoptosis in beta cell lines [13], [17] but only limited decrease in viability in human islets [17], have no impact on rat islet viability [13], or can improve rat islets viability in the presence of cytokines [13].

A link between an alteration of the UPS and the progression of diabetes has never been demonstrated. Indeed, proteasome dysfunction correlates with obesity in humans [10], [18], [19], and kidneys from diabetic Akita rats or streptozotocin-injected mice show a decrease in chymotrypsin-like activity and an increase in polyubiquinated proteins [20], but this is not seen in others tissus. Pancreatic islets from Zucker diabetic rats [21] or human diabetic obese donors [22] also exhibit an increased level of polyubiquitinated proteins, suggesting a default in the elimination of damaged/misfolded proteins, but the implication of a proteasome activity default is lacking. A recent study shows a decreased in islets proteasome activity of type 2 diabetic patients [23] together with a decrease of UPS proteins transcription and expression. However, whether hyperglycemic environment is deleterious for beta cells through an alteration of the proteasome is still unknown.

In this aim, we studied the importance of proteasome dysfunction as a potential molecular mechanism associated with beta cell failure in glucotoxic conditions, in the INS-1E beta cell line and in primary rat and human islets. We chose to explore the role of ER-stress in this process. Finally, we investigated if this decreased proteasome activity can be pharmacologically corrected through the use of GLP-1 analogs. Our results clearly show that proteasome activity is a key determinant of the survival/apoptosis balance in beta cells, that could be part of the glucotoxicity mechanisms involved in diabetes pathophysiology.

## Materials and Methods

### Ethics statement

Male diabetic Goto-Kakizaki (GK) rats and Wistar control rats were obtained from the Paris colony (GK/Par), and maintained at the University Paris-Diderot animal core (Agreement A-75-13-17). The experimental protocol was approved by the institutional Animal Care and Use Ethical Committee of the Paris-Diderot University (registration number CEEA-40), in accordance with accepted standards of animal care as established in the French National Center for Scientific Research (CNRS) guidelines.

Human pancreas were harvested from brain-dead non-diabetic donors, identified from the CRISTAL register of the Agence de la Biomedecine, which inventories all graft recipients in France. Informed consent from the donor family and agreement for scientific research was obtained through the Departement de la Coordination Hospitalière de Prélèvements d'Organes of Montpellier Hospital. Experiments were performed in agreement with the Institutional Ethical Committee of the French Agence de la Biomedecine (ref. PFS13-008).

### Cell culture

INS-1E beta cells were originally cloned and characterized [24] by Pr. P. Maechler from the parental rat INS-1 cell line in the Laboratory of Pr. C.B. Wollheim (CMU, University of Geneva, Switzerland). INS-1E cells (a kind gift from Pr. P. Maechler) were cultured between passages 55-90 in RPMI-1640 medium containing 10 mM glucose and 5% FCS (InVitrogen, St-Aubin, France)[24]. Cells were grown for four days in RPMI-1640 to reach 75% confluency, then incubated for 48 hours in RPMI-1640 medium with glucose ranging from 10 to 33 mM, in the presence of BSA 0.5% instead of FCS in order to sensitize cells to glucotoxicity [25]. Proteasome inhibitors MG-132, ALLN, lactacystin (Calbiochem, VWR, France) or Bortezomib (Velcade® Janssen, France) were added during the last 16 hours. Exendin-4 was from Bachem (Bubendorf, Switzerland). Following treatments, cells were washed in PBS and harvested in lysis buffer (see composition in [26]) for Western-blot analysis, or in proteasome lysis solution (20 mM TRIS pH 7.5, 0.1 mM EDTA, 20% glycerol, 0.05% Nonidet-P40, 1 mM mercapto-ethanol, and 1 mM ATP) for proteasome activities measurement.

### Animals

Twelve-week-old male non-diabetic Wistar and diabetic Goto-Kakizaki (GK) rats were maintained at constant temperature (21–23°C) with a 12∶12 hr light/dark cycle. Food and water were available *ad libitum*. Rat pancreatic islets were isolated as previously described [27] and groups of 300–500 islets were stored at −80°C until studied. Islets were harvested in similar lysis buffer than INS-1E for Western-blot analysis or proteasome activities measurement.

### Human pancreatic islets processing

Human islets were isolated from four brain-dead non-diabetic donors at the Laboratory for Diabetes Cell Therapy (Montpellier, France) or the Cell Isolation and Transplantation Center (Geneva, Switzerland) according to a modified version of the automated method [28], [29]. After isolation, islets were cultured for recovery during 1–5 days in CMRL-1066 medium (InVitrogen) containing 5.6 mM glucose, 10% FBS, antibiotics and glutamine [15]. For experiments with proteasome inhibitors, islets were then cultured during 16 h in RPMI-1640 medium containing 5.6 mM glucose and 1% human albumin (LFD Biomedicaments, Les-Ulis, France), and treated like rat islets. For chronic high-glucose experiments, islets were cultured for 14 days in RPMI-1640 medium containing 5.6 or 33.3 mM glucose, in the absence of FCS but the presence of 0.4 mM palmitate bound to BSA (0.5% BSA, molar ratio palmitate/BSA  = 5∶1) in order to sensitize islets to glucotoxicity [30].

### Western-blot analysis

Following treatments, INS-1E cells, rat or human islets were lysed for 30 min at 4°C in a cold lysis buffer as described above, then rapidly sonicated and centrifuged at 12,000 g for 20 min. Supernatants were denatured by boiling them in Laemmli's sample buffer, normalized for protein content by a BCA assay, and equal amounts of proteins (25 μg of protein/lane) resolved by 10% SDS-PAGE. After immunoblotting on nitrocellulose membranes, membranes were cut up into 3 or 4 pieces according to the molecular weights profile, in order to observe simultaneously different proteins on the same blot. Each piece was incubated with primary then secondary antibodies, and proteins were visualized by chemiluminescence detection, as previously described [15]. Band densities were quantitatively analyzed by Image J (NIH, USA) and normalized to actin, used as our loading control. Anti-cleaved caspase-3, anti-PARP (Poly-ADP-ribose-polymerase), anti-ubiquitin, anti-CHOP (C/EBP-homologous protein), and anti-rabbit IgG antibodies were from Cell-Signaling (Ozyme, St-Quentin-en-Yvelines, France). Anti-proteasome 20S-β5 subunit and anti-mouse IgG antibodies were from Santa-Cruz (Tebu-bio, Le-Perray-en-Yvelines, France). Anti-β-actin was from Sigma-Aldrich (St-Quentin-Fallavier, France).

### Proteasome activities measurement

Following treatments, INS-1E cells and islets were lysed and processed for proteasome activity as described [31]. After rapid sonication, lysates were centrifuged (12,000 g) and supernatants collected. After proteins level equalization, 5 μg supernatants were combined with substrate buffer (50 mM HEPES pH 7.5, 5 mM EGTA) and 50 μM of each proteasome substrate; Z-ARR-MCA, Suc-LLVY-MCA, and Z-LLE-MCA (Calbiochem) were used to measure trypsin-like, chymotrypsin-like, and caspase-like activities, respectively. The three proteasome activities were distinguished from background proteolytic activities by using a proteasome inhibitor that nearly totally inhibit fluorescence changes. The fluorescence intensity was measured at 380 nm excitation and 460 nm emission with a Flexstation3 Reader (Molecular Devices, France) using free MCA as standard. Proteasomal activities were expressed as arbitrary units.

### Apoptosis and cell death detection

Apoptosis was investigated using immunoblot by evaluating cleavage of caspase-3 into a 17/19 kDa fragment corresponding to the active pro-apoptotic form of caspase-3, and cleavage of PARP (a caspase-3 target involved in DNA repair and cell survival) into a 89 kDa fragment. Total cell death was evaluated using the Cell Death Detection ELISA^PLUS^ kit (Roche), as per the manufacturer's instructions, using 20 μl of culture supernatant. Due to our prolonged treatments (≥48 h), DNA fragments will be present in the supernatant of cells culture as the result of the lysis of late apoptotic cells as well as necrotic cells. Absorbance was measured at 405 nm using a Mithras LB940 Reader (Berthold, Thoiry, France) and the results expressed in arbitrary units of oligonucleosome-associated histone.

### Statistical analysis

All data are presented as mean ± SEM for n independent experiments. Statistical differences between groups were analyzed by Student's *t*-test or by ANOVA followed by the Tukey's multiple comparison test where appropriate, using Graphpad-Prism software (La Jolla, USA). A *P* value <0.05 was considered significant.

## Results

### Chronic exposure of INS-1E cells to high-glucose affects proteasome activity and ubiquitination

**Figure 1:**
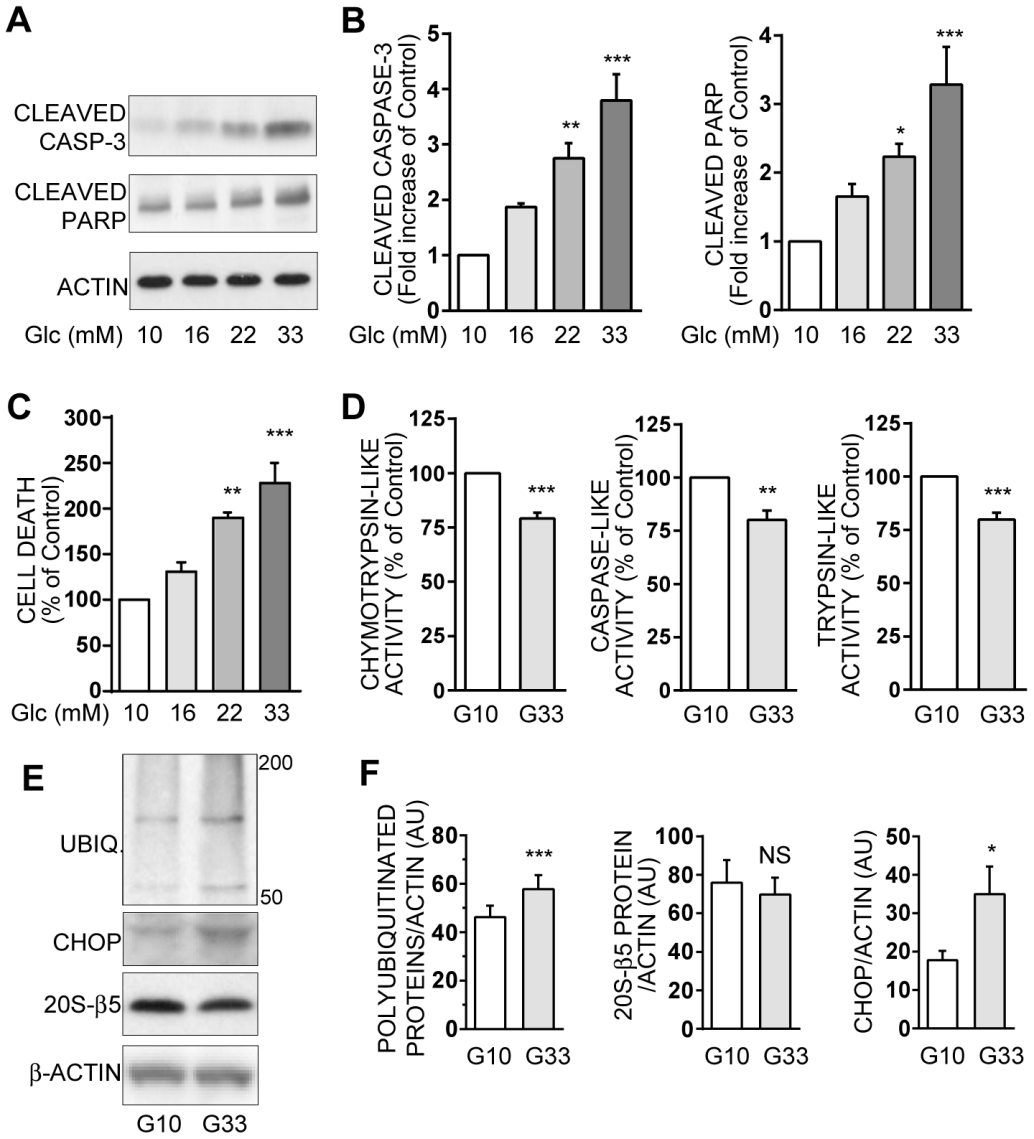
Chronic high glucose induces apoptosis and proteasome activities decrease in INS-1E cells. INS-1E cells were cultured for 48 hours at increasing concentrations of glucose ranging from 10 mM (G10) to 33 mM (G33). **A**: Protein levels of cleaved caspase-3, cleaved PARP and actin were analyzed by Western blotting in INS-1E cells exposed to different glucose concentrations. Actin was used as a loading control. Immunoblots presented are representative of 5 independent experiments. **B**: Quantitative analysis of bands densities of Western blot (as presented in A) for cleaved caspase-3 and cleaved PARP were normalized to actin. Results are presented as means ± SEM of 5 independent experiments and expressed as fold increase compared to the G10 value. **C**: Total cell death was measured in the culture supernatants of INS-1E cells after 48 hours. Results are presented as means ± SEM of 4 independent experiments and expressed as percentage of the G10 value. **D**: Chymotrypsin-like, caspase-like, and trypsin-like activities were measured in lysates from G10- or G33-exposed INS-1E cells. Results are presented as means ± SEM of 6 independent experiments and expressed as percentage of the G10 value. **E**: Levels of polyubiquitinated proteins, CHOP protein -an endoplasmatic reticulum stress marker-, 20S-β5 protein -a proteasome subunit-, and actin were analyzed by Western blottin in INS-1E cells after 48 hours of culture either in 10 mM or 33 mM glucose. Actin was used as a loading control. Immunoblots presented are representative of 4 independent experiments. **F**: Quantitative analysis of bands densities of Western blots (as presented in E) were normalized to actin. Results are presented as means ± SEM of 4 independent experiments and expressed in arbitrary unit (AU). *P<0.05, **P<0.01, and ***P<0.001.

In INS-1E cells, increasing glucose from an optimal (10 mM) to a supra-physiologic (33 mM) level during 48 h is deleterious and leads to dose-dependent increases in cleaved-caspase-3, cleaved-PARP (Figures 1A and 1B), and total cell death (Figure 1C). Besides, this chronic exposure to high-glucose significantly decreases the 3 proteasome activities, with a 20–25% loss of the chymotrypsin-like, caspase-like, and trypsin-like activities (Figure 1D). In parallel, the polyubiquitinated proteins level is increased by 26% in the presence of high glucose, whereas the 20S-β5 proteasome subunit level is not significantly altered (Figures 1E and 1F). Finally, we confirm that endoplasmic reticulum (ER) stress, as evidenced by the two fold increase in CHOP expression (Figures 1E and 1F), is involved in the increased apoptosis observed in beta cells submitted to high glucose.

### Impaired proteasome activities in hyperglycemic GK rat islets

**Figure 2:**
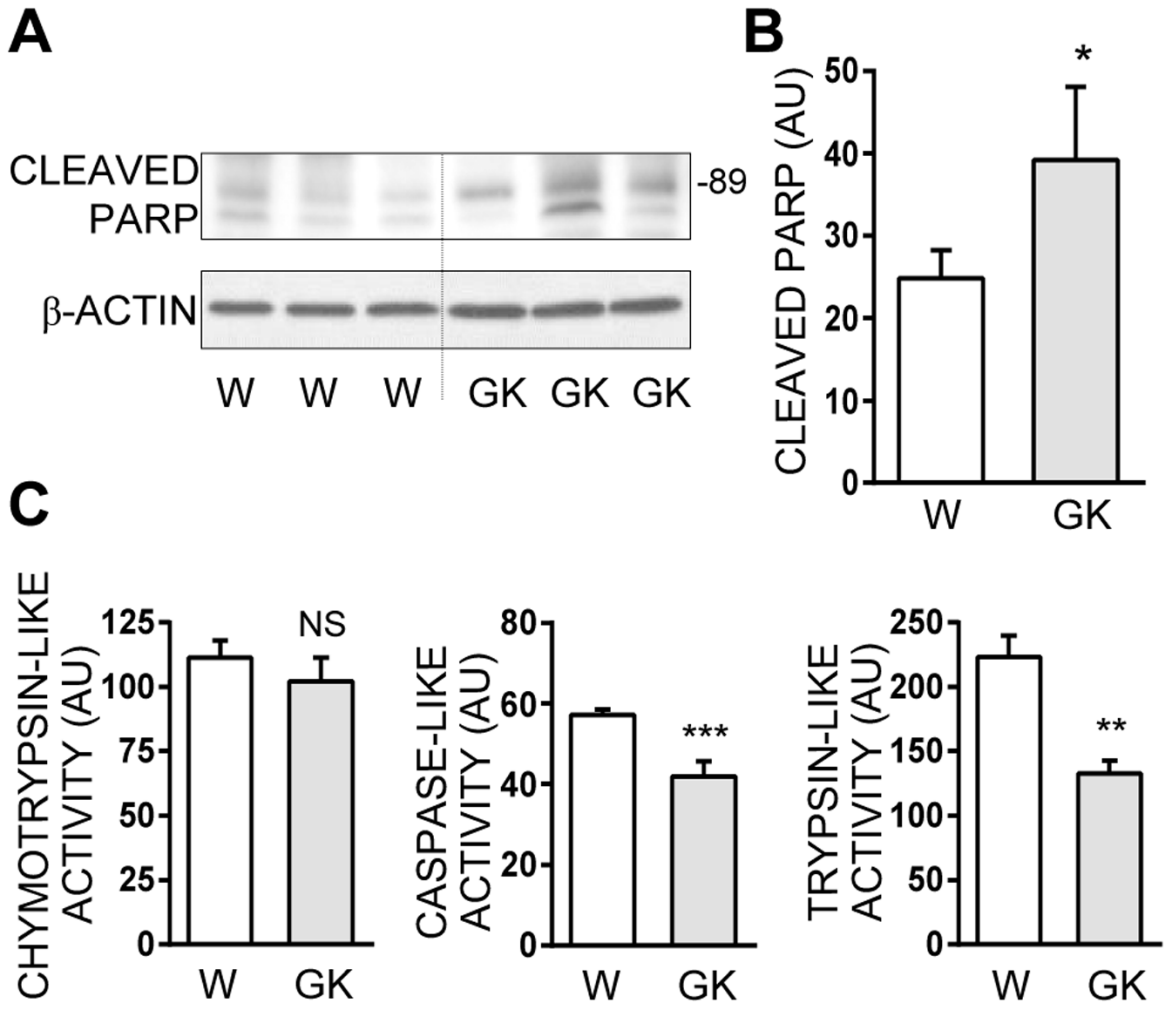
Islets from hyperglycemic GK rats exhibit decreased proteasome activities together with increased cleaved PARP levels. **A**: Cleaved PARP protein levels were analyzed by Western blotting in 9 Wistar rats (W) or 5 GK rats (GK) independent islets preparations. Three most representative immunoblots from Wistar and GK rats are presented and actin was used as a loading control. **B**: Quantitative analysis of bands densities for cleaved PARP obtained by Western blot from 9 Wistar rats (W) or 5 GK rats (GK) independent islets preparations (as shown in A) were normalized to actin. Results are presented as means ± SEM and are expressed in arbitrary units (AU). **C**: Chymotrypsin-like, caspase-like, and trypsin-like activities were measured in the lysates of islets from Wistar or GK rats. Results are presented as means ± SEM of 9 (W) or 5 (GK) independent islets preparations and are expressed in arbitrary unit (AU). *P<0.05 and **P<0.01 and ***P<0.001.

We assess *ex vivo* the impact of a hyperglycemic environment on beta cell proteasome function using the GK rat diabetic model [32], [33]. Pancreatic islets from 5 GK rats exhibiting mild hyperglycemia (around 9.0 mM) are compared to islets from 9 Wistar control rats exhibiting normoglycemia (around 5.0 mM). GK rats islets show a slight increase in apoptosis, as revealed by PARP cleavage (Figures 2A and 2B). More importantly, GK rat islets display a 25% reduction in caspase-like activity (p<0.01), a 40% reduction in trypsin-like activity (p<0.01), whereas chymotrypsin-like activity was not significantly decreased (−10%, p = 0.20) (Figure 2C). This suggests that the hyperglycemic environment could be linked to decreased proteasome activities *in vivo*.

### Loss of proteasome activity leads to apoptosis in INS-1E cells

**Figure 3:**
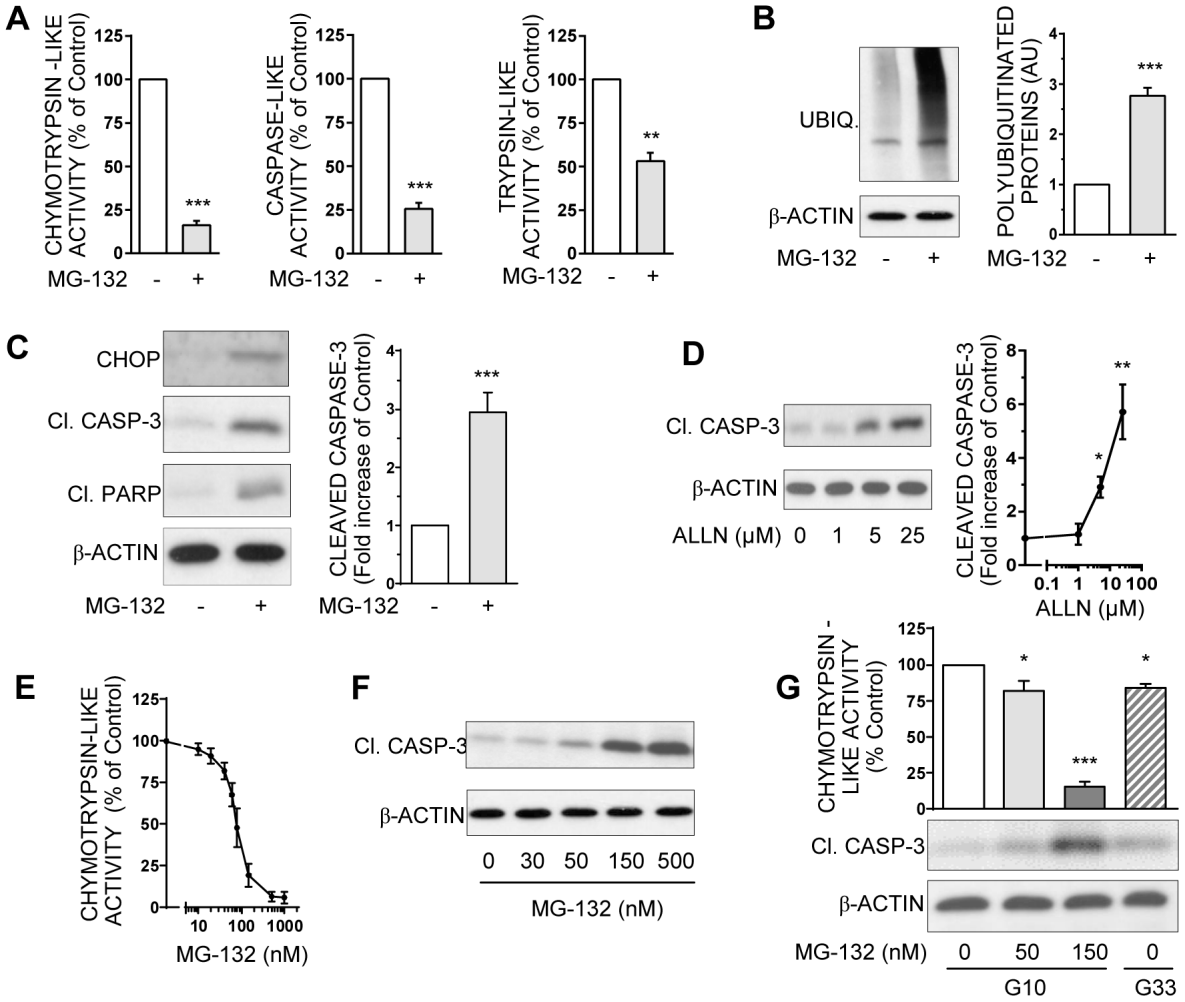
Pharmacological inhibition of proteasome induces polyubiquitinated proteins accumulation and caspase-dependent apoptosis in INS-1E cells. **A**: Chymotrypsin-like, caspase-like, and trypsin-like activities were measured in the lysates of INS-1E cells treated or not with 150 nM MG-132. Results are presented as means ± SEM of 5 independent experiments and expressed as percentage of the value without MG-132. **B**: Levels of polyubiquitinated proteins were analyzed by Western blotting in INS-1E cells treated or not with 150 nM MG-132 and quantified. Results shown as immunoblots are representative from 5 independent experiments. Quantitative analysis of global membrane densities from immunoblots normalized to actin is presented. Results are presented as means ± SEM and expressed as fold induction of the value without MG-132. **C**: Levels of cleaved caspase-3, cleaved PARP, CHOP, and actin were analyzed by Western blotting in INS-1E cells exposed or not to 150 nM MG-132. Results presented are representative immunoblots and quantitative analysis of bands densities normalized to actin from 5 independent experiments. Results are means ± SEM and expressed as fold increase of the value without MG132. **D**: Protein levels of cleaved caspase-3 and actin were analyzed in INS-1E cells exposed or not to increasing concentrations (1–25 μM) of the UPS inhibitor ALLN by Western blotting, and were quantified. Immunoblots presented are representative of 3 independent experiments. Quantitative analysis of bands densities of Western blot for cleaved caspase-3 normalized to actin is presented as means ± SEM. Results are expressed as fold increase of the control value without ALLN. **E**: Chymotrypsin-like activity was measured in the lysates from INS-1E cells treated or not with increasing concentrations of MG-132 (10 nM to 1 μM). Results are presented as means ± SEM of 3 independent experiments and expressed as a percentage of the value without MG-132. **F**: Protein levels of cleaved caspase-3 and actin were analyzed by Western blotting in INS-1E cells exposed or not to increasing concentrations (30–500 nM) of the UPS inhibitor MG-132. Immunoblots presented are representative of 3 independent experiments. **G**: INS-1E cells were cultured either in 10 mM glucose with increasing concentrations of MG-132 (0,50 and 150 nM) or in high (33 mM) glucose. Chymotrypsin-like activity was measured in cells lysates and presented as means ± SEM. Protein levels of cleaved caspase-3 and actin were analyzed by Western blotting. Data are representative of 3 independent experiments. *P<0.05, **P<0.01, and ***P<0.001.

In order to implicate a proteasome dysfunction in beta cell apoptosis, we treated INS-1E cells with the widely used proteasome inhibitor (PI) MG-132. In cells cultured into optimal glucose concentration for survival (i.e. 10 mM), MG-132 (150 nM) induces a 90, 80 and 50% inhibition of chymotrypsin-like, caspase-like, and trypsin-like activities, respectively (Figure 3A). This is accompagnied by a significant increase (+150%) in polyubiquitinated proteins level (Figure 3B), whereas the expression of proteasome subunit 20S-β5 is not modified (data not shown). In parallel, MG-132 induces the emergence of a caspase-dependent apoptosis as revealed by the cleavage of caspase-3 and PARP (Figure 3C). The implication of ER stress in the apoptotic effect of MG-132 is confirmed by the emergence of CHOP expression (Figure 3C).

The pro-apoptotic effect of PIs is also found using the structurally different inhibitors ALLN (Figure 3D) and lactacystin (data not shown). Moreover, increasing MG-132 concentration from 10 to 1000 nM gradually inhibits chymotrypsin-like activity (Figure 3E). This sigmoidal-shaped effect shows a low Ki at 50 nM and a near-total inhibition of UPS activity around 1 μM MG-132. This gradual decrease in proteasome activity is concommittant with an increase in cleaved caspase-3 (Figure 3F), definitely linking proteasome dysfunction and apoptosis. Finally, a 50 nM MG-132-exposure, leading to a minimal loss of proteasome activity about 20%, is sufficient to induce apoptosis (Figure 3G). Interestingly, chymotrypsin-like activity and apoptotic level are similarly affected in INS-1E cells exposed either to low dose (50 nM) of MG-132 or to a chronic high-glucose level (Figure 3G). Thus, slight impairment of UPS activity in beta cells induced similar apoptosis than high-glucose exposure.

### Chronic high-glucose induces apoptosis and proteasome dysfunction in human islets

**Figure 4:**
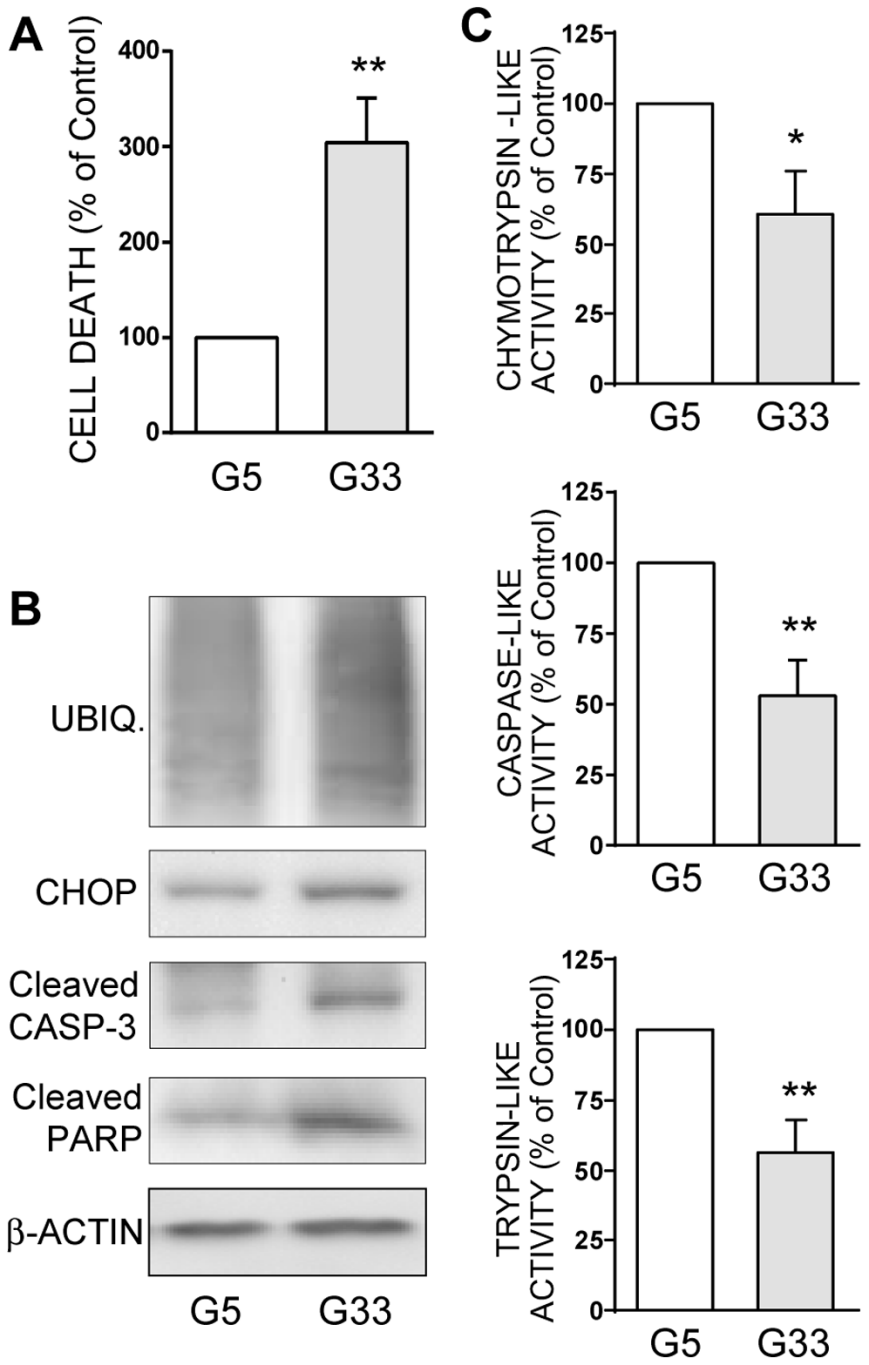
A chronic high glucose treatment decreases proteasome activities and increases apoptosis in human islets. Human islets were cultured for 14 days in RPMI medium containing an optimal (5.6 mM, G5) or a high (33 mM, G33) glucose concentration. Four independent experiments were conducted using human islets from four independent islets isolations (4 different donors). **A**: Total cell death was measured in the supernatants of culture medium. Data are expressed as means ± SEM as a percentage of the G5 value. **B**: Levels of polyubiquitinated proteins, CHOP, cleaved caspase-3, cleaved PARP, and actin were analyzed by Western blotting after 14 days of culture. Immunoblots presented are representative of the 4 independent experiments. **C**: Chymotrypsin-like, caspase-like, and trypsin-like activities were measured in the lysates from human islets after 14 days of culture. Data presented are means ± SEM and are expressed as as a percentage of G5. *P<0.05, **P<0.01.

In human islets, a 14 days-chronic high glucose (33 mM) exposure clearly induces a three-fold increase in total cell death (Figure 4A) and an increase in apoptosis as seen by caspase-3 and PARP cleavage (Figure 4B). More importantly, all the three proteasome activities were significantly reduced by 40–50% in high glucose-exposed islets (Figure 4C). Finally, polyubiquitinated proteins and CHOP expressions increase in these glucotoxic conditions (Figure 4B), thus confirming the presence of an ER stress linked to UPS dysfunction in human islets.

### Loss of proteasome activity leads to apoptosis in human islets

**Figure 5:**
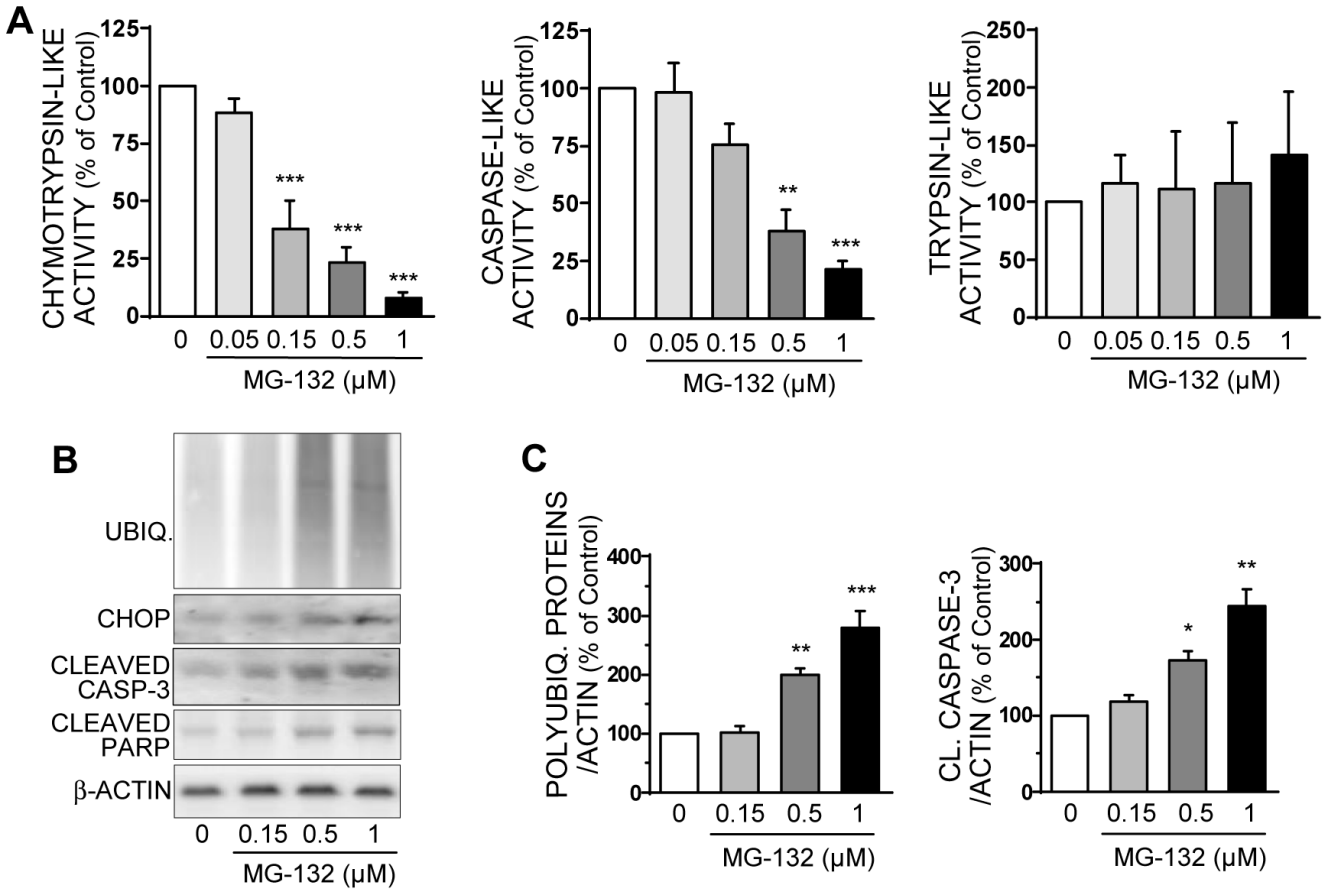
Pharmacologic inhibition of proteasome activity in human islets increases apoptosis. Human islets were treated for 16-132 at increasing concentrations from 50 nM to 1 μM. Four independent experiments were conducted using human islets from four independent islets isolations (4 different donors). **A**: Chymotrypsin-like, caspase-like, and trypsin-like activities were measured in the lysates of human islets treated or not with MG-132. Results are presented as means ± SEM and expressed as a percentage of the value without MG-132. **B**: Levels of polyubiquitinated proteins, CHOP, cleaved caspase-3, cleaved PARP, and actin were analyzed by Western blotting in human islets after 16 hours of culture. Immunoblots presented are representative of the 4 independent experiments. **C**: Quantitative analysis of bands densities of Western blots as presented in B were normalized to actin. Results are expressed as a percentage of the value without MG-132. *P<0.05, **P<0.01, and ***P<0.001.

In human islets, MG-132 (0.05–1 μM) induces a progressive decrease in chymotrypsin-like and caspase-like activities, without significantly affecting trypsin-like activity (Figure 5A). In parallel, MG-132 dose-dependently increases the expression of polyubiquitinated proteins, CHOP, cleaved caspase-3 and cleaved PARP (Figure 5B and 5C). These effects are also found with 5 μM lactacystin or 100 nM bortezomib (data not shown). Thus PIs, by reducing proteasome activities, clearly induce polyubiquitinated proteins accumulation and the emergence of a caspase-dependent apoptotic program through the promotion of ER-stress in rodent beta cells and human islets.

### Chronic exposure of INS-1E cells to high-glucose induces hypersensitivity to proteasome inhibitors

**Figure 6:**
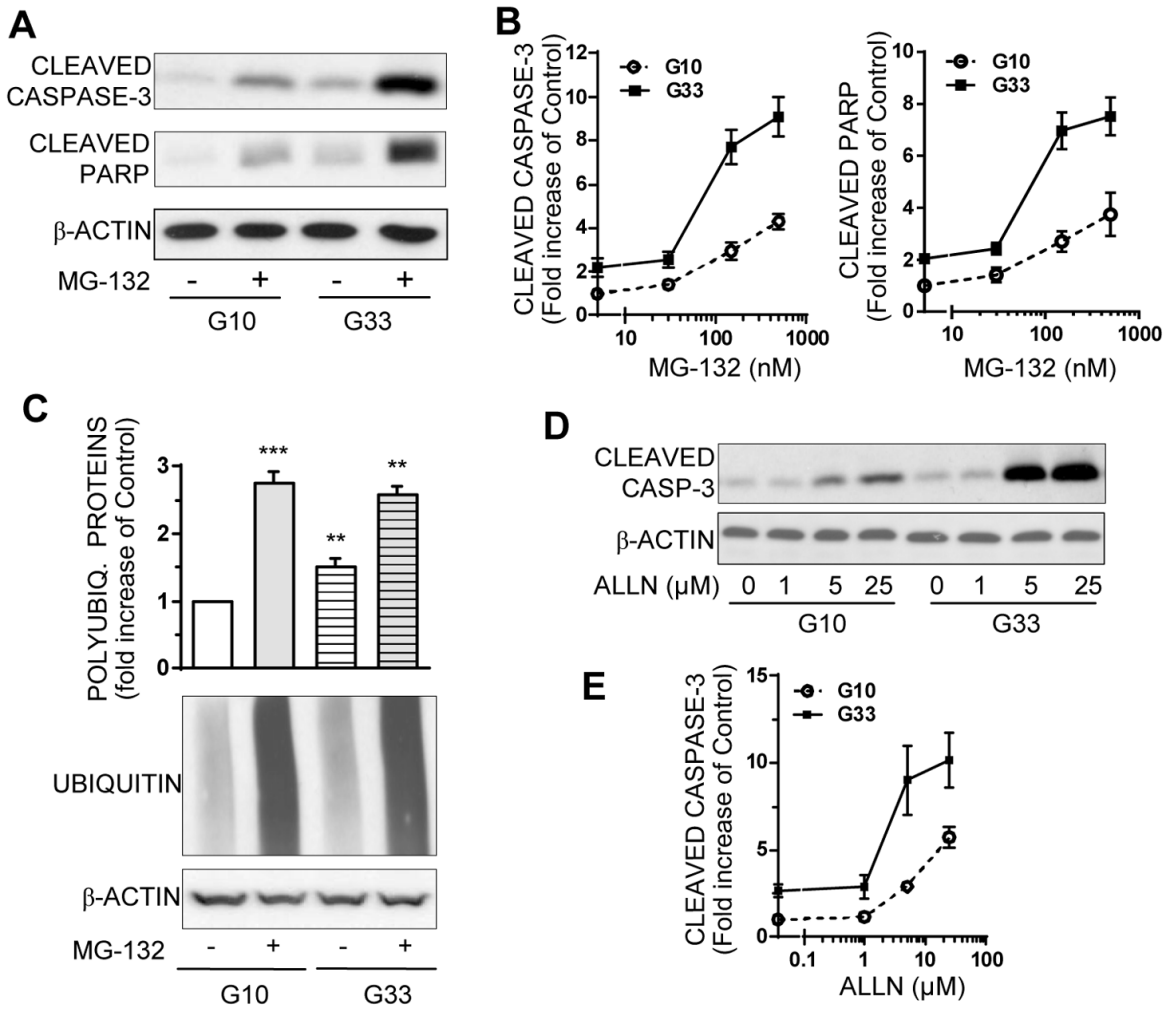
Chronic high-glucose exposure induces INS-1E cell hypersensitivity to the proapoptotic effect of proteasome inhibitors. INS-1E Cells were cultured for 48 hours with an optimal 10 mM (G10) or a high 33 mM (G33) glucose concentration, and treated with proteasome inhibitors MG-132 or ALLN for the last 16 h. **A**: Levels of cleaved caspase-3, cleaved PARP, and actin were analyzed by Western blotting in INS-1E cells cultured with or without150 nM MG-132 at normal or high glucose concentrations. Immunoblots presented are representative of 3 independent experiments. **B**: Quantitative analysis of bands densities normalized to actin from immunoblots as shown in A detecting cleaved caspase 3 or cleaved PARP in cells treated with 30, 150 or 500 nM MG-132 at optimal or high glucose concentrations. Results are presented as means ± SEM from 3 independent experiments and are expressed as fold increase compared to the value in cells cultured at optimal glucose (G10) without MG-132. **C**: INS-1E cells were exposed to 0 or 150 nM MG-132 at optimal (G10) or high glucose (G33) concentrations. Protein levels of polyubiquitinated proteins and actin were analyzed by Western blotting and quantified. Quantitative analysis of bands densities for polyubiquitinated proteins were normalized to actin. Results are presented as means ± SEM of 3 independent experiments and expressed as fold increase over the G10 value without MG-132. **D**: INS-1E cells were treated with another proteasome inhibitor ALLN at increasing concentrations (1–25 μM) and at normal or high glucose concentrations. Protein levels of cleaved caspase-3 and actin were analyzed by Western blotting. Immunoblots presented are representative of 3 independent experiments. **E**: Quantitative analysis of cleaved caspase-3 bands densities from immunoblots as shown in (D) were normalized to actin. Results are presented as means ± SEM of 3 independent experiments and expressed as fold increase compared to the G10 value without ALLN. *P<0.05, **P<0.01, ***P<0.001.

We further investigated the role of combination of high-glucose exposure and proteasome inhibitors on beta cells. While 150 nM MG-132 induces a slight but significant apoptosis in INS-1E cells cultured in 10 mM glucose medium (Figure 6A), this same concentration of MG-132 induces a drastic apoptosis in INS-1E cells cultured at high-glucose, as evidenced by the 8-fold increase of cleaved caspase-3 and cleaved PARP (Figure 6B). However, the polyubiquitinated proteins level induced by MG-132 is similarly high in normal or high-glucose concentrations (Figure 6C) as if a ceiling was reached. Similar results on apoptosis are obtained with ALLN (Figure 6D and 6E) or lactacystin (data not shown). This drastic increase of apoptosis by proteasome inhibitors coupled to high glucose culture reveals hypersensitivity of beta cells to UPS inhibition in glucotoxic conditions.

### Exendin-4 prevents proteasome activity dysfunction and protects INS-1E cells from apoptosis

**Figure 7:**
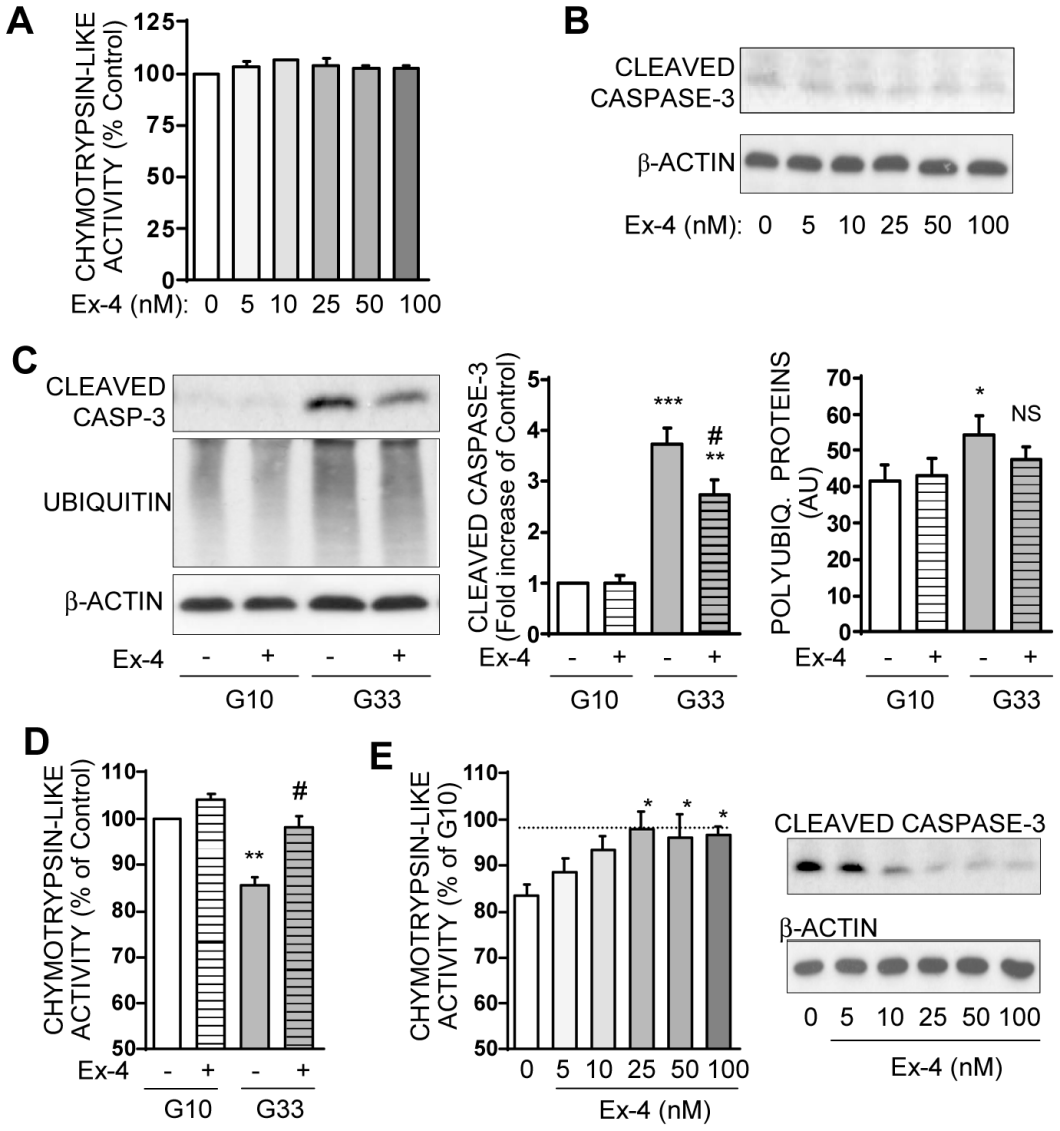
Exendin-4 partially prevents chronic high glucose-induced apoptosis and proteasome dysfunction in INS-1E cells. **A**: INS-1E cells were cultured in 10 mM glucose medium for 48 hours with increasing concentrations (5–100 nM) of Exendin-4. Chymotrypsin-like activity was measured in cells lysates at the end of the treatment. Results are presented as means ± SEM of 3 independent experiments and expressed in percentage of the value without Exendin-4. **B**: Protein levels of cleaved caspase-3 levels and actin were analyzed by Western blotting in INS-1E cells cultured in 10 mM glucose medium for 48 hours with increasing concentrations (5–100 nM) of Exendin-4. Immunoblots presented are representatives of 3 independent experiments. **C**: Levels of cleaved caspase-3, polyubiquitinated proteins, and actin were analyzed by Western blotting in cells treated for 48 hours with 25 nM Exendin-4 in 10 mM (G10) or 33 mM glucose (G33) medium. Immunoblots presented are representative of 6 independent experiments. Quantitative analysis of bands density normalized to actin from these 6 independent experiments is also shown. Results are presented as means ± SEM and expressed as fold increase compared to the G10 value without Exendin-4. *P<0.05; **P<0.01, ***P<0.001 vs. G10; #P<0.05 vs. G33. **D**: Chymotrypsin-like activity was measured in lysates from INS-1E cells treated for 48 hours with 25 nM Exendin-4 in 10 mM (G10) or 33 mM glucose (G33) medium. Results are presented as means ± SEM of 3 independent experiments and expressed as the percentage of the G10 value. **P<0.01 vs. G10; #P<0.05 vs. G33. **E**: Chymotrypsin-like activity was measured in cells cultured for 48 hours in 33 mM glucose medium and with increasing concentrations of Exendin-4 (5–100 nM). Protein levels of cleaved caspase-3 and actin were analyzed by Western blotting. Immunoblots presented are representatives of 3 independent experiments. *P<0.05 vs G33 without Exendin-4.

We finally investigated whether pharmacological agents that activate PKA in beta cells, such as the GLP-1 analog Exendin-4, could positively interact with proteasome activities. First, in INS-1E cells cultured into optimal 10 mM glucose medium, Exendin-4 does not significantly modify the chymotrypsin-like activity (Figure 7A), nor the others proteasome activities (data not shown) or the cleaved caspase-3 expression (Figure 7B).

Then, in INS-1E cells cultured in the presence of chronic high-glucose (33 mM), we observe, as expected, an increased caspase-dependent apoptotic program, decreased UPS activities, and accumulation of polyubiquitinated proteins (Figure 7C and 7D). In these glucotoxic conditions, Exendin-4 (25 nM) clearly improves cell survival, with a significant reduction in the emergence of cleaved caspase-3 induced by high-glucose (−36%, p<0.05) (Figure 7C). Of importance, Exendin-4 also prevents proteasome dysfunction induced by high-glucose as shown on chymotrypsine-like activity (Figure 7D) and other proteasome activities (data not shown). Noteworthily, Exendin-4 tends to decrease (p = 0.13 vs. G33 alone) the polyubiquitinated levels observed in the presence of chronic G33 (Figure 7C). Finally, this protective effect of Exendin-4 is dose-dependent on both proteasome activity and cell survival, reaching an optimum at 25 nM (Figure 7E).

## Discussion

The balance between survival and death in pancreatic beta cells plays a central role in the pathogenesis of diabetes. We report here that a chronic exposure of rat beta cells or human islets to high-glucose concentrations leads to a decrease in the proteasome activity and to an accumulation of polyubiquitinated proteins. This inhibition of UPS activity leads to beta cell apoptosis in a dose-dependent manner. Moreover, pharmacological inhibition of UPS in the context of glucotoxicity drastically increases these apoptotic effects, drawing the concept of hypersensitization of beta cells to proteasome inhibitors (PIs). UPS activity -in this context of glucotoxicity- can be partially restored by Exendin-4.

First, we demonstrate that glucotoxic conditions can alter proteasome activities. This major finding must be connected to a previous study [20] showing that proteasome activities were decreased in endothelial cells cultured in high-glucose medium, and in kidney cells from diabetic mice but not in other tissues. We show here that this cell type-specific phenomenon can also affect beta cell population. Accumulation of polyubiquitinated proteins has been previously observed in several models of diabetic beta cells [21], [22], suggesting indirectly an UPS alteration in hyperglycemic environment. However, authors attributed this accumulation either 1) to a default in the autophagy system [21], or 2) to a deficiency in the deubiquitinating enzyme UCH-L1 [22]. Our results clearly indicate that a decrease in proteasome activity is also implicated in the accumulation of polyubiquitinated proteins observed in beta cells submitted to a glucotoxic environment.

When studying proteasome function in beta cells, special attention should be paid to the specific proteasome activity observed. Here we observed that the inhibitory effect of MG-132 on trypsin-like activity is less important in INS-1E cells and totally absent in human islets compared to the chymotrypsin-like and caspase-like activities. Indeed proteasomes are very heterogeneous protein complexes: differences exists in the molecular assembly of the catalytic active β subunits, as well as differences in their posttranslational modifications and in the presence of distinct associating partners [34]. Thus, each cell contains a specific set of proteasome subtypes, exhibiting specific proteasomal activities that strongly vary between tissues and age in the same animal, and express different susceptibilities to proteasome inhibitors such as MG-132 [35]. In this context, it was very recently noticed that trypsin-like activity shows the greatest interspecies difference [36]. Finally, in both INS-1E and human islets, the decrease of two of the three proteasome activities in the presence of MG-132 is enough to induce a global decrease of proteasome activity as observed with the increase in polyubiquitinated protens level.

The other main point of this study is that the inhibition of proteasome activity can have *per se* a deleterious impact on beta cell survival. Noteworthily, this pro-apoptotic effect of PIs exists even for a slight −20%- reduction of proteasome activity, the same percentage of inhibition induced by high-glucose culture or 50 nM MG-132. Our results are in accordance with previous studies showing that high doses of PIs reduce viability of clonal MIN6 and INS-1E beta cells [13], [17]. For entire islets, the literature data were controversial, as a decrease in viability was observed in human islets cultured with epoxomycin [17], whereas lactacystin had no impact on beta cell viability of young rats [13]. We confirm here that immortalized cell lines are more sensitive to the pro-apoptotic effect of PIs than primary cells, even if the latter can still be impacted by higher dose of PIs [37].

We show that inhibition of proteasome activity in beta cells could be a new link between glucotoxicity and apoptosis. This phenomenon -via genetic predisposition or epigenetic regulation- may thus exist in diabetic patients, participating in beta cell dysfunction. Indeed, Bugliani *et al*. [23] recently showed that UPS genes and activity were particularly altered in beta cells of type 2 diabetic patients. Our study underlines the direct effect of high-glucose on UPS dysfunction, at a molecular level (probably at a post-traductional level) and not, as suggested in the latter study, at a transcriptional level. This is compatible with the hypothesis of Queisser *et al*. [20] suggesting that glycation of the 20S-β5 proteasome subunit by high-glucose treatment could affect its activity. Further studies should be done to confirm this hypothesis.

Endoplasmic reticulum and oxidative stresses are widely known to be the main mechanisms of glucotoxicity [38], [39], even if cytokine production [40] or hypoxia also represent others putative stressors (review in [2]). Besides, the role of proteasome inhibition in glucotoxicity is poorly defined. Indeed, the relationships between ER and proteasome are complex since they can influence each other. In our study, UPS dysfunction directly participates in triggering ER stress, CHOP expression, caspase cleavage and apoptosis onset. This results confirms previous studies showing that proteasome inhibition by MG-132 or bortezomib can induce ER stress, thus activating caspases cleavage and apoptosis in cancerous or primary epithelial cells [41], [42]. However, the opposite is also true since Menendez-Benito *et al*. [43] first observed in a panel of UPS reporter cell lines as well as in an *in vivo* transgenic mice model that ER stress could have an inhibitory effect on the UPS, especially inducing a subtle, slow and progressive decrease in proteasome activity, leading to a “compromised UPS”. In brief, proteasome inhibition by MG-132 or high-glucose exposure could induce ER stress and apoptosis, but ER stress alone could also induce a progressive proteasome inhibition in parallel to caspase-dependent apoptosis. Taken together, this suggests that the glucotoxic-induced proteasome dysfunction observed in our study could be placed both above and below ER stress in the cascade leading from chronic hyperglycemia to beta cell apoptosis and diabetes.

UPS dysfunction and proteasome activity inhibition can promote neurodegenerative diseases such as Huntington and Alzheimer diseases, both characterized by protein misfolding, aggregates accumulation, and ER stress increase. Diabetes is often compared to them because of islet amyloidosis, supposed to be due to the UPS dysfunction [22]. Here, our study clearly reinforces the parallel between diabetes and neurodegenerative diseases through the demonstration of the role of UPS dysfunction and ER stress in beta cells exposed to high glucose.

Depending on situations and cell types, UPS may play more than a simple role of garbage collector and can direct cell function and survival. In lymphocytes, glioma cells or hematopoietic progenitor cells, UPS is placed at the top of the apoptotic machinery, upstream of the mitochondrial and caspase activation [44], [45]. Our results support a similar central role of UPS in the beta cell apoptosis machinery. Indeed, in our study, glucotoxic conditions - mainly via ER and oxidative stresses- added to decreased proteasome activity with PIs led to a drastic apoptosis. This observation is compatible with the recent concept of sensitization already described on myeloma cells cultured with PIs and other ER stressors [46]. The sensitivity of myeloma cells to apoptosis induction by PIs varies greatly between cell types and correlates with expression levels of proteasome subunits and/or the proteasome activity itself, as a result of a balance between proteasome workload and degradation capacity [46]. Similarly, high-glucose-treated beta cells display a decrease in proteasome activities together with an increased workload in insulin synthesis and secretion. In glucotoxic conditions, it seems thus logical that UPS inhibition can lead to an over-workload, majored ER stress, and drastic apoptosis. That confirms that the maintenance of UPS integrity is a key player in the misfolded protein clearance and in the regulation of essential functions of beta cells. This beta cell hypersensitivity to PIs under glucotoxicity could have clinical implications, suggesting hypersensitivity of diabetic patients to proteasome inhibitors like bortezomib used in myeloma. Indeed, PIs efficacy may be reinforced on cancerous cells, but their adverse effects -peripheral neuropathy or anemia- may be more numerous and severe in diabetic patients [47].

Finally, the demonstration that the GLP-1-receptor agonist Exendin-4 preserves proteasome activity from the deleterious effects of chronic high glucose reinforces the fact that the preservation of UPS activity is crucial for beta cell survival, especially in glucotoxic conditions. Moreover, a parallel exists between Exendin-4 dose-responses on proteasome activity increase and caspase-3 cleavage reduction. This restoration of UPS activity by a GLP-1 analogue can be explained by the role of PKA in beta cells. Indeed, GLP-1 analogues act through PKA in beta cells and PKA has been shown to be a direct activator of proteasome activity [48], [49]. This opens the possibility to increase *in vivo* the activity of GLP-1 analogues with activators of UPS.

In conclusion our work integrates UPS as a new key player in the mechanism of glucotoxicity in beta cells. We report here that chronic high-glucose exposure decreases proteasome activities in beta cells, leading to UPS dysfunction. Proteasome dysfunction could be part of the glucotoxic mechanisms through inducing ER stress and apoptosis. Apoptotic hypersensitization of beta cells exists when pre-existing glucotoxicity and UPS inhibition are added: this creates a vicious circle resulting in increased apoptosis of beta cells. GLP-1 analogues can help stopping this vicious circle. Our results suggest that proteasome function constitutes an important and underestimated target for restoring pancreatic beta cell survival and GLP-1 analogues efficacy.
