# Correction: Proteasome Dysfunction Mediates High Glucose-Induced Apoptosis in Rodent Beta Cells and Human Islets

**DOI:** 10.1371/journal.pone.0102652

**Published:** 2014-07-07

**Authors:** Christophe Broca, Elodie Varin, Mathieu Armanet, Cécile Tourrel-Cuzin, Domenico Bosco, Stéphane Dalle, Anne Wojtusciszyn

After publication of our work, it has been noticed that some control actin panels, used to confirm similar protein loading across the Western blot gel, were mistakenly used at the wrong place during final assembly of illustrations. Please note that control actin panels have been rectified in Figure 1A, Figure 3, Figures 6A&D and Figures 7B&E. The original raw blots used for these illustrations are provided with the revised figures. The other blots and histograms are unchanged. We would like to thank the reader who noticed this mistake. We apologize for this error and refer readers to the corrected Figures 1, 3, 6 and 7 provided in this Correction.

**Figure 1:**
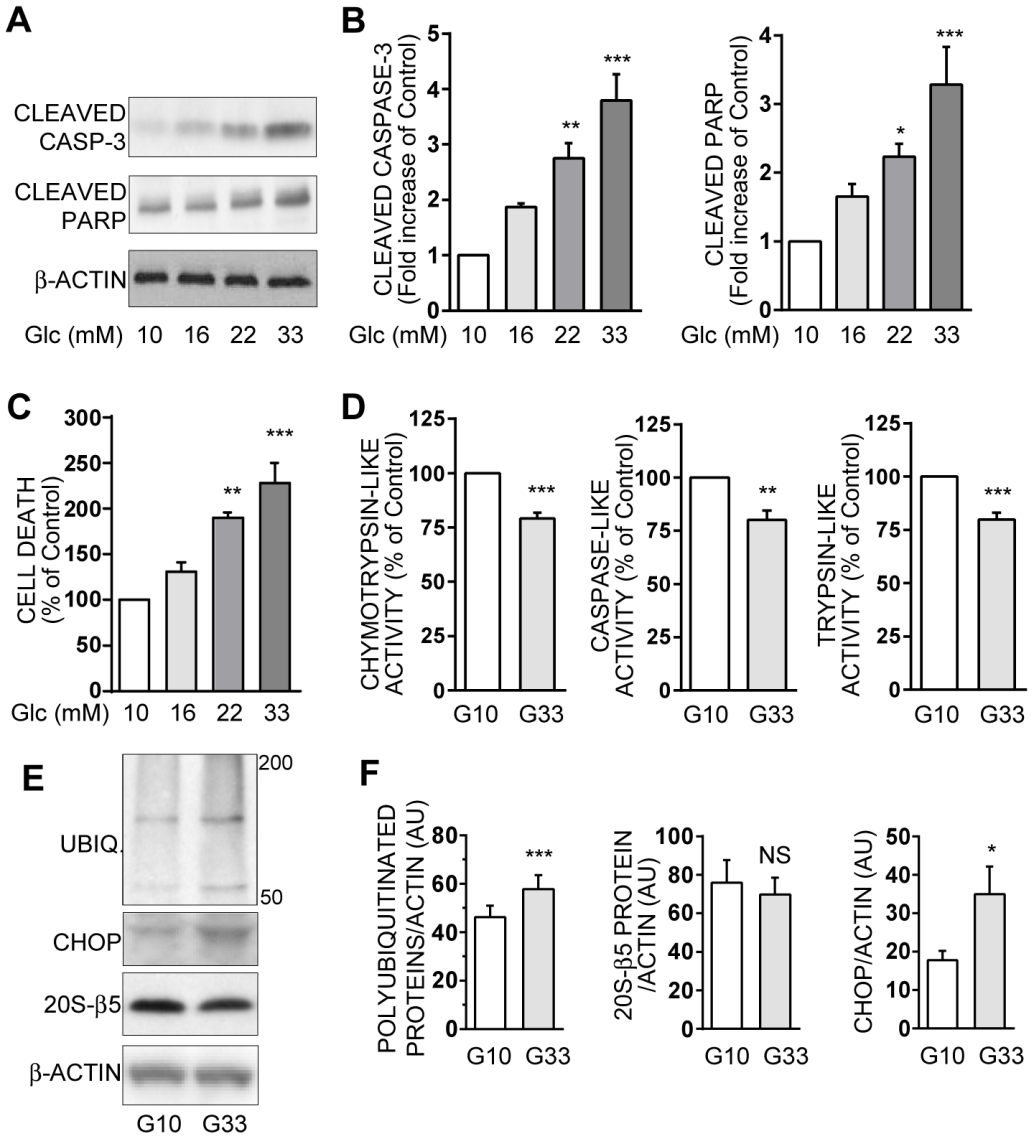
Chronic high glucose induces apoptosis and proteasome activities decrease in INS-1E cells. INS-1E cells were cultured for 48 hours at increasing concentrations of glucose ranging from 10 mM (G10) to 33 mM (G33). **A**: Protein levels of cleaved caspase-3, cleaved PARP and actin were analyzed by Western blotting in INS-1E cells exposed to different glucose concentrations. Actin was used as a loading control. Immunoblots presented are representative of 5 independent experiments. **B**: Quantitative analysis of bands densities of Western blot (as presented in A) for cleaved caspase-3 and cleaved PARP were normalized to actin. Results are presented as means ± SEM of 5 independent experiments and expressed as fold increase compared to the G10 value. **C**: Total cell death was measured in the culture supernatants of INS-1E cells after 48 hours. Results are presented as means ± SEM of 4 independent experiments and expressed as percentage of the G10 value. **D**: Chymotrypsin-like, caspase-like, and trypsin-like activities were measured in lysates from G10- or G33-exposed INS-1E cells. Results are presented as means ± SEM of 6 independent experiments and expressed as percentage of the G10 value. **E**: Levels of polyubiquitinated proteins, CHOP protein -an endoplasmatic reticulum stress marker-, 20S-β5 protein -a proteasome subunit-, and actin were analyzed by Western blottin in INS-1E cells after 48 hours of culture either in 10 mM or 33 mM glucose. Actin was used as a loading control. Immunoblots presented are representative of 4 independent experiments. **F**: Quantitative analysis of bands densities of Western blots (as presented in E) were normalized to actin. Results are presented as means ± SEM of 4 independent experiments and expressed in arbitrary unit (AU). *P<0.05, **P<0.01, and ***P<0.001.

**Figure 3:**
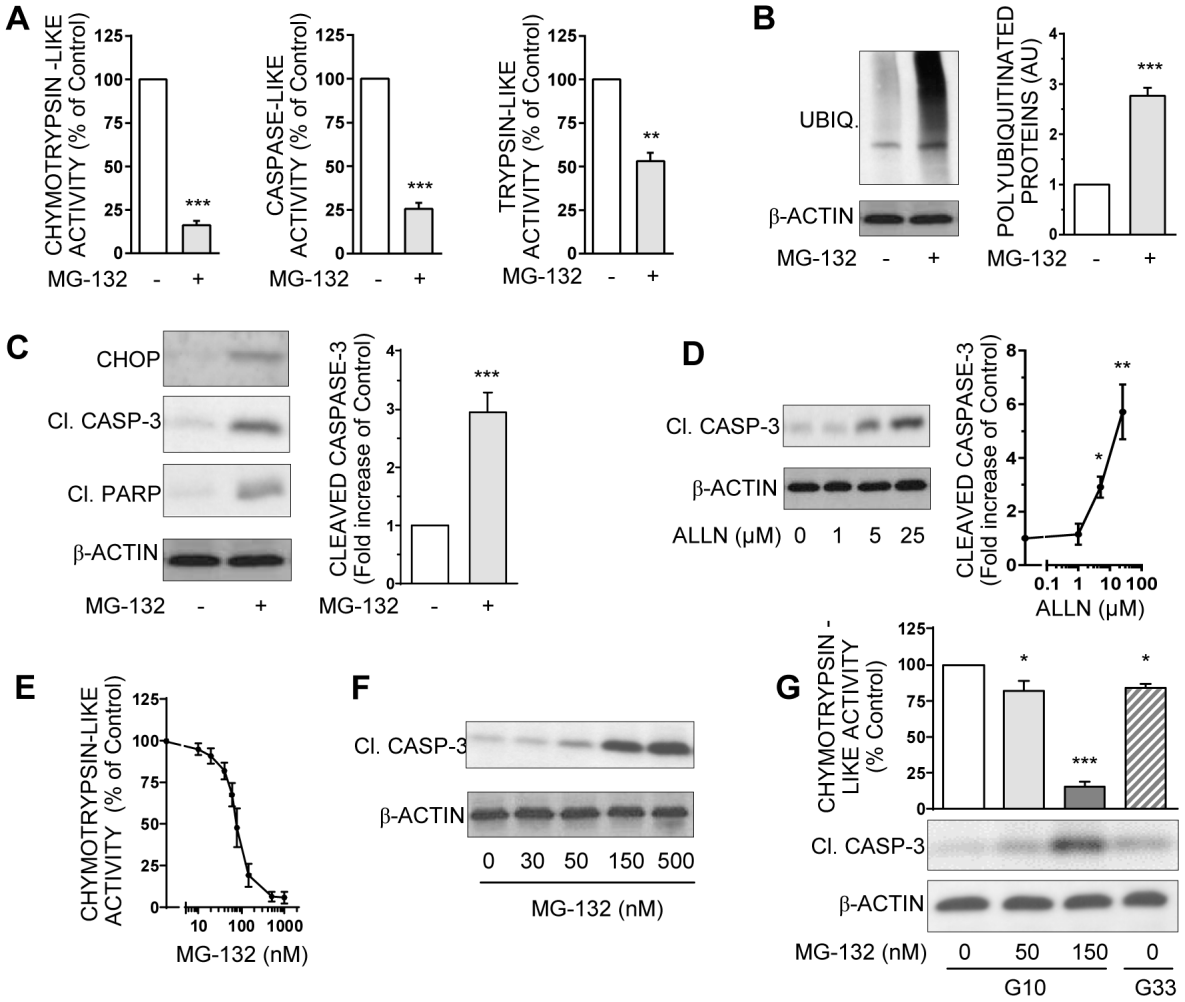
Pharmacological inhibition of proteasome induces polyubiquitinated proteins accumulation and caspase-dependent apoptosis in INS-1E cells. **A**: Chymotrypsin-like, caspase-like, and trypsin-like activities were measured in the lysates of INS-1E cells treated or not with 150 nM MG-132. Results are presented as means ± SEM of 5 independent experiments and expressed as percentage of the value without MG-132. **B**: Levels of polyubiquitinated proteins were analyzed by Western blotting in INS-1E cells treated or not with 150 nM MG-132 and quantified. Results shown as immunoblots are representative from 5 independent experiments. Quantitative analysis of global membrane densities from immunoblots normalized to actin is presented. Results are presented as means ± SEM and expressed as fold induction of the value without MG-132. **C**: Levels of cleaved caspase-3, cleaved PARP, CHOP, and actin were analyzed by Western blotting in INS-1E cells exposed or not to 150 nM MG-132. Results presented are representative immunoblots and quantitative analysis of bands densities normalized to actin from 5 independent experiments. Results are means ± SEM and expressed as fold increase of the value without MG132. **D**: Protein levels of cleaved caspase-3 and actin were analyzed in INS-1E cells exposed or not to increasing concentrations (1–25 µM) of the UPS inhibitor ALLN by Western blotting, and were quantified. Immunoblots presented are representative of 3 independent experiments. Quantitative analysis of bands densities of Western blot for cleaved caspase-3 normalized to actin is presented as means ± SEM. Results are expressed as fold increase of the control value without ALLN. **E**: Chymotrypsin-like activity was measured in the lysates from INS-1E cells treated or not with increasing concentrations of MG-132 (10 nM to 1 µM). Results are presented as means ± SEM of 3 independent experiments and expressed as a percentage of the value without MG-132. **F**: Protein levels of cleaved caspase-3 and actin were analyzed by Western blotting in INS-1E cells exposed or not to increasing concentrations (30–500 nM) of the UPS inhibitor MG-132. Immunoblots presented are representative of 3 independent experiments. **G**: INS-1E cells were cultured either in 10 mM glucose with increasing concentrations of MG-132 (0,50 and 150 nM) or in high (33 mM) glucose. Chymotrypsin-like activity was measured in cells lysates and presented as means ± SEM. Protein levels of cleaved caspase-3 and actin were analyzed by Western blotting. Data are representative of 3 independent experiments. *P<0.05, **P<0.01, and ***P<0.001.

**Figure 6:**
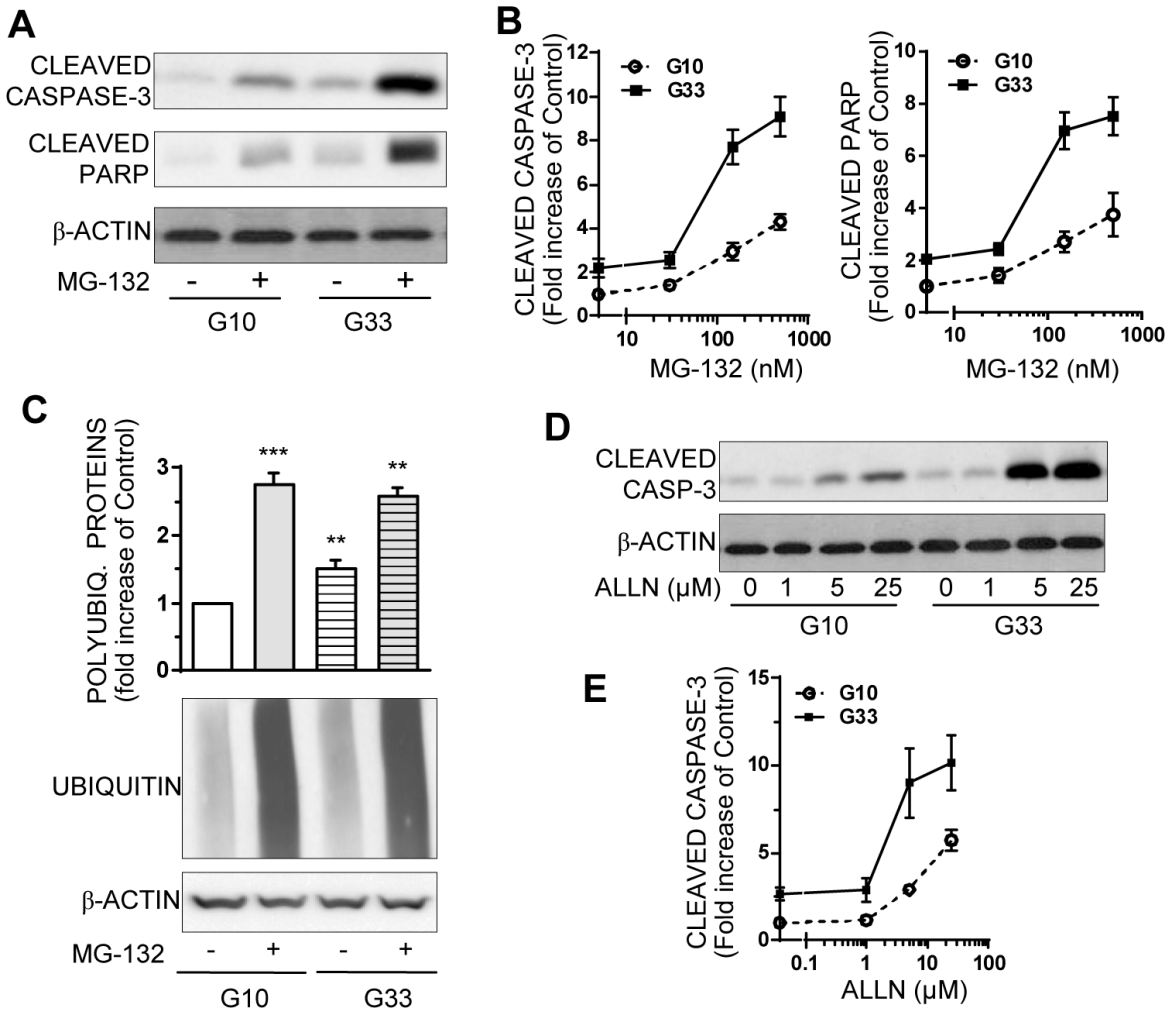
Chronic high-glucose exposure induces INS-1E cell hypersensitivity to the proapoptotic effect of proteasome inhibitors. INS-1E Cells were cultured for 48 hours with an optimal 10 mM (G10) or a high 33 mM (G33) glucose concentration, and treated with proteasome inhibitors MG-132 or ALLN for the last 16 h. **A**: Levels of cleaved caspase-3, cleaved PARP, and actin were analyzed by Western blotting in INS-1E cells cultured with or without150 nM MG-132 at normal or high glucose concentrations. Immunoblots presented are representative of 3 independent experiments. **B**: Quantitative analysis of bands densities normalized to actin from immunoblots as shown in A detecting cleaved caspase 3 or cleaved PARP in cells treated with 30, 150 or 500 nM MG-132 at optimal or high glucose concentrations. Results are presented as means ± SEM from 3 independent experiments and are expressed as fold increase compared to the value in cells cultured at optimal glucose (G10) without MG-132. **C**: INS-1E cells were exposed to 0 or 150 nM MG-132 at optimal (G10) or high glucose (G33) concentrations. Protein levels of polyubiquitinated proteins and actin were analyzed by Western blotting and quantified. Quantitative analysis of bands densities for polyubiquitinated proteins were normalized to actin. Results are presented as means ± SEM of 3 independent experiments and expressed as fold increase over the G10 value without MG-132. **D**: INS-1E cells were treated with another proteasome inhibitor ALLN at increasing concentrations (1–25 µM) and at normal or high glucose concentrations. Protein levels of cleaved caspase-3 and actin were analyzed by Western blotting. Immunoblots presented are representative of 3 independent experiments. **E**: Quantitative analysis of cleaved caspase-3 bands densities from immunoblots as shown in (D) were normalized to actin. Results are presented as means ± SEM of 3 independent experiments and expressed as fold increase compared to the G10 value without ALLN. *P<0.05, **P<0.01, ***P<0.001.

**Figure 7:**
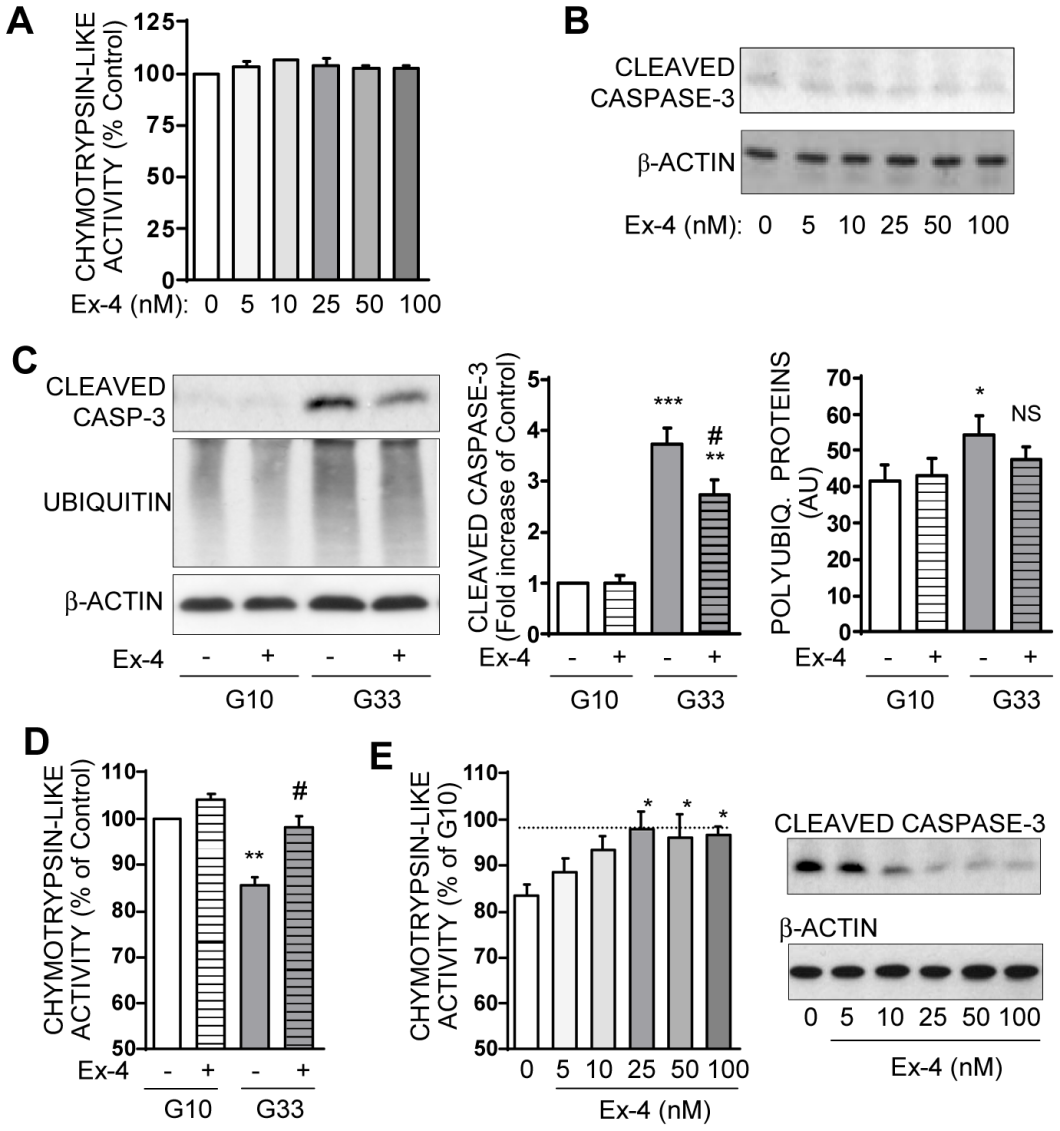
Exendin-4 partially prevents chronic high glucose-induced apoptosis and proteasome dysfunction in INS-1E cells. **A**: INS-1E cells were cultured in 10 mM glucose medium for 48 hours with increasing concentrations (5–100 nM) of Exendin-4. Chymotrypsin-like activity was measured in cells lysates at the end of the treatment. Results are presented as means ± SEM of 3 independent experiments and expressed in percentage of the value without Exendin-4. **B**: Protein levels of cleaved caspase-3 levels and actin were analyzed by Western blotting in INS-1E cells cultured in 10 mM glucose medium for 48 hours with increasing concentrations (5–100 nM) of Exendin-4. Immunoblots presented are representatives of 3 independent experiments. **C**: Levels of cleaved caspase-3, polyubiquitinated proteins, and actin were analyzed by Western blotting in cells treated for 48 hours with 25 nM Exendin-4 in 10 mM (G10) or 33 mM glucose (G33) medium. Immunoblots presented are representative of 6 independent experiments. Quantitative analysis of bands density normalized to actin from these 6 independent experiments is also shown. Results are presented as means ± SEM and expressed as fold increase compared to the G10 value without Exendin-4. *P<0.05; **P<0.01, ***P<0.001 vs. G10; #P<0.05 vs. G33. **D**: Chymotrypsin-like activity was measured in lysates from INS-1E cells treated for 48 hours with 25 nM Exendin-4 in 10 mM (G10) or 33 mM glucose (G33) medium. Results are presented as means ± SEM of 3 independent experiments and expressed as the percentage of the G10 value. **P<0.01 vs. G10; #P<0.05 vs. G33. **E**: Chymotrypsin-like activity was measured in cells cultured for 48 hours in 33 mM glucose medium and with increasing concentrations of Exendin-4 (5–100 nM). Protein levels of cleaved caspase-3 and actin were analyzed by Western blotting. Immunoblots presented are representatives of 3 independent experiments. *P<0.05 vs G33 without Exendin-4.

## Supporting Information

Figure S1 Raw Blots for Figures 1A, 3B, 3C, & 3D.
(TIF)

Figure S2 Raw Blots for Figures 3F, 6A, & 6D.
(TIF)

Figure S3 Raw Blots for Figures 7B & 7E.
(TIF)
