# Correction: Correction: Proteasome Dysfunction Mediates High Glucose-Induced Apoptosis in Rodent Beta Cells and Human Islets

**DOI:** 10.1371/journal.pone.0293491

**Published:** 2023-10-23

**Authors:** The PLOS ONE Editors

## Notice of republication

This article was republished on August 31, 2023, to correct for technical errors that affected viewing Figs 1, 3, 6 and 7 in the online version of the article. There were no changes to the text of the article or the PDF. The publisher apologizes for the error.Reference
