# Supplementary figures and images for: Correction: Proteasome Dysfunction Mediates High Glucose-Induced Apoptosis in Rodent Beta Cells and Human Islets
Source: PLoS One. 2014 Jul 7;9(7):e102652. doi: 10.1371/journal.pone.0102652 (PMC4085027; doi:10.1371/journal.pone.0102652)

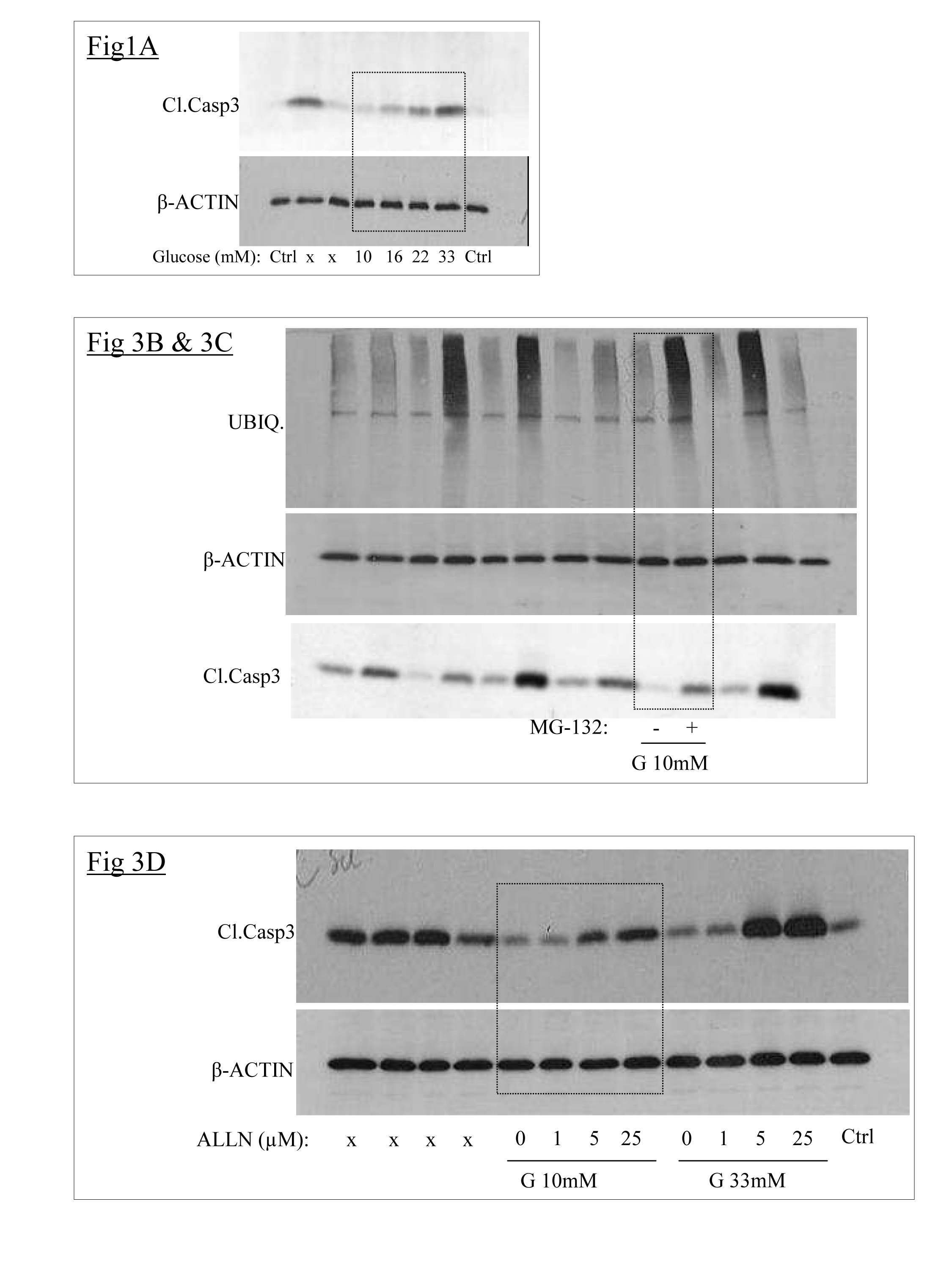

Supplement: Figure S1 — Raw Blots for Figures 1A, 3B, 3C, & 3D. (TIF) [file pone.0102652.s001.tif]

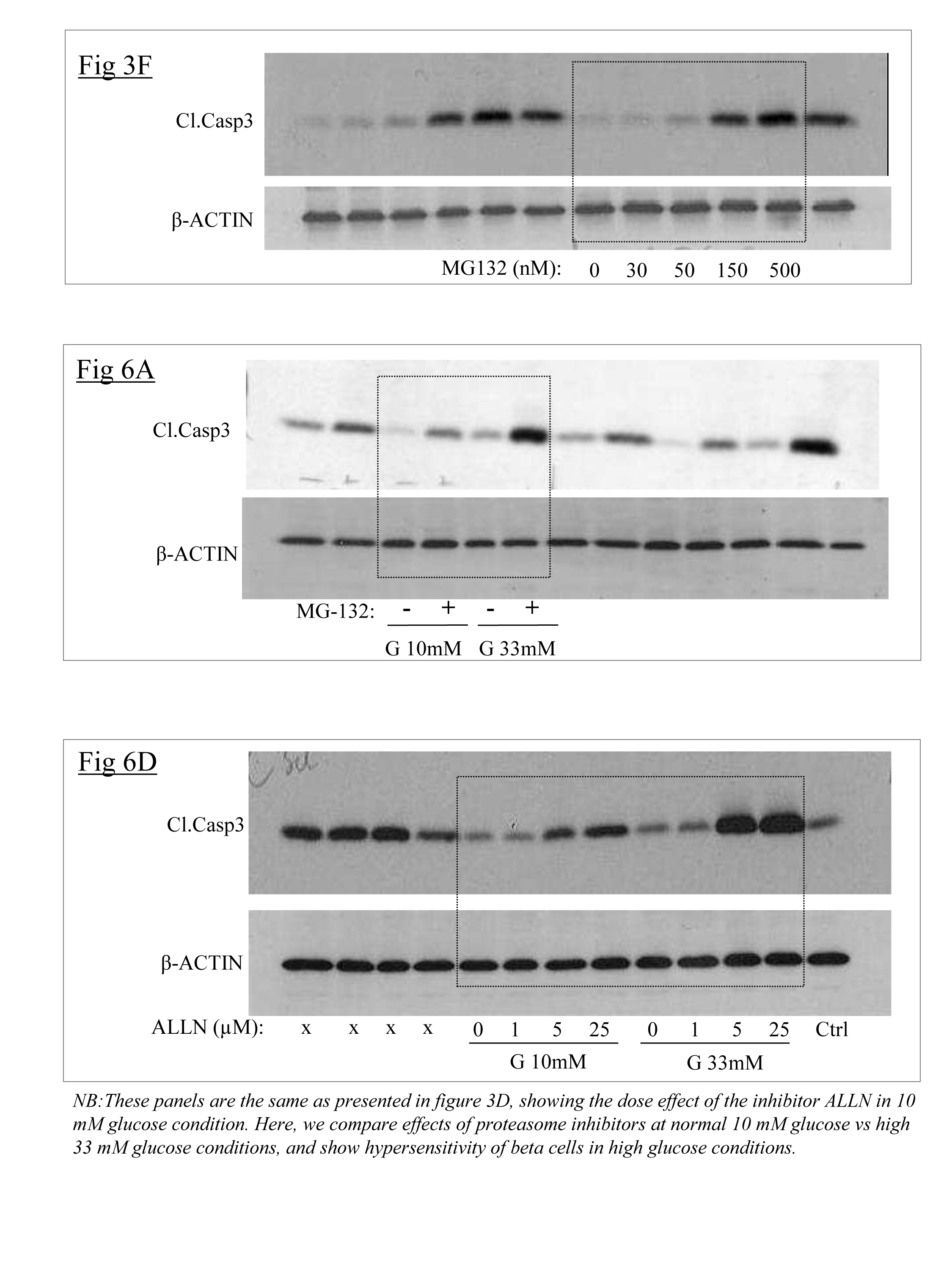

Supplement: Figure S2 — Raw Blots for Figures 3F, 6A, & 6D. (TIF) [file pone.0102652.s002.tif]

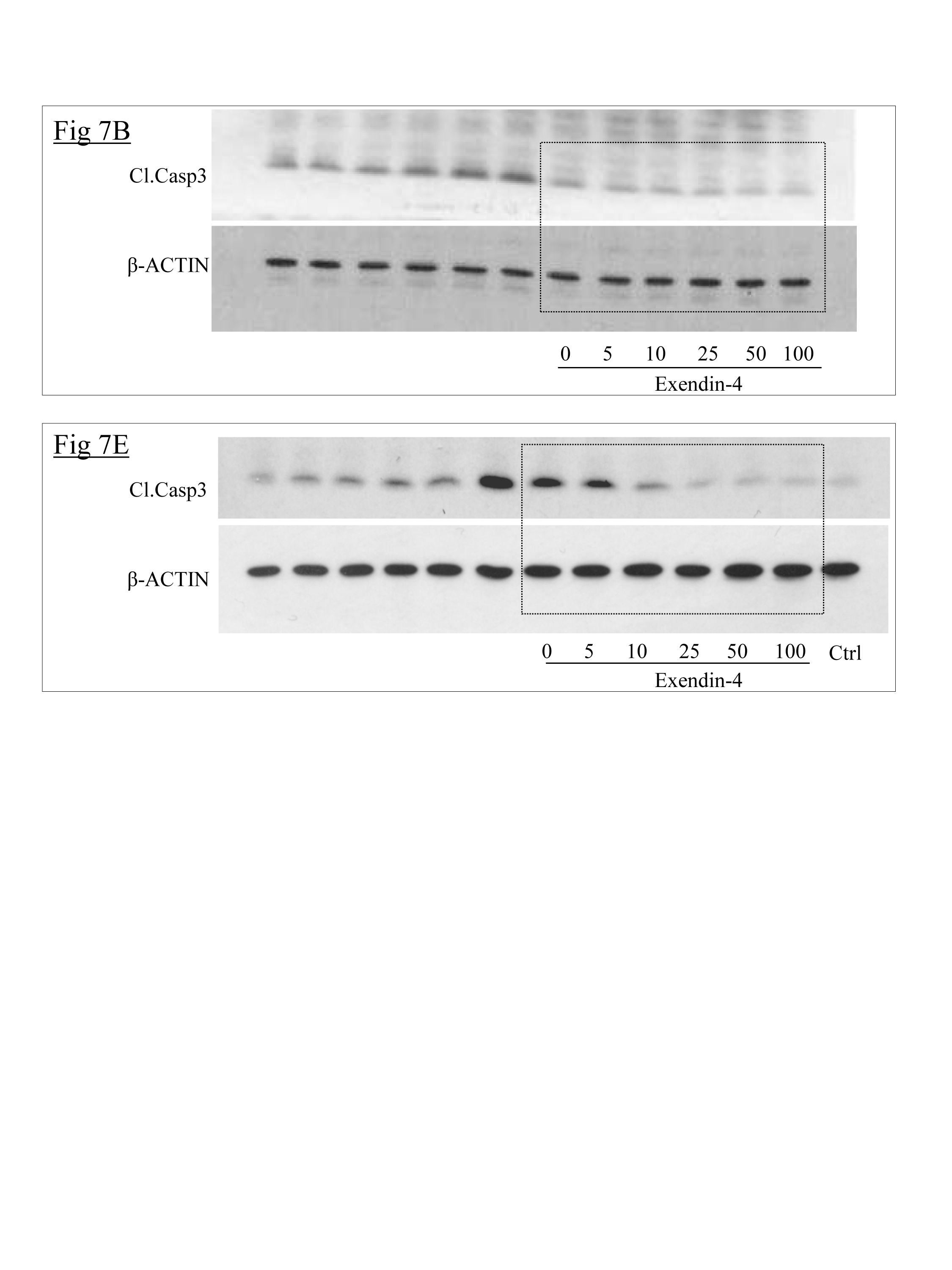

Supplement: Figure S3 — Raw Blots for Figures 7B & 7E. (TIF) [file pone.0102652.s003.tif]
